# The nutrition toolbox permits *in silico* generation, analysis, and optimization of personalized diets through metabolic modelling

**DOI:** 10.1093/bioadv/vbaf325

**Published:** 2026-01-08

**Authors:** Bram Nap, Bronson Weston, Annette Brandt, Maximilian F Wodak, Ina Bergheim, Ines Thiele

**Affiliations:** School of Medicine, University of Galway, Galway H91 TK33, Ireland; Digital Metabolic Twin Centre, University of Galway, Galway H91 TK33, Ireland; School of Medicine, University of Galway, Galway H91 TK33, Ireland; Department of Nutritional Sciences, R.F. Molecular Nutritional Science, University of Vienna, Vienna 1090, Austria; Department of Nutritional Sciences, R.F. Molecular Nutritional Science, University of Vienna, Vienna 1090, Austria; Department of Nutritional Sciences, R.F. Molecular Nutritional Science, University of Vienna, Vienna 1090, Austria; School of Medicine, University of Galway, Galway H91 TK33, Ireland; Digital Metabolic Twin Centre, University of Galway, Galway H91 TK33, Ireland; Discipline of Microbiology, University of Galway, Galway H91 TK33, Ireland; Ryan Institute, University of Galway, Galway H91 TK33, Ireland; APC Microbiome Ireland, Cork T12 K8AF, Ireland

## Abstract

**Motivation:**

Nutrition is an important factor in human health, used to alleviate or prevent symptoms of various diseases. However, the effects of nutrition on the gut microbiome and human metabolism are not well understood. Whole-body metabolic models (WBMs) have been applied to study relationships between regional diets and human/microbiome metabolism. This method requires diets to be defined at the metabolite level, rather than the food item level, which has gated the application of personalized diets to WBMs.

**Results:**

We developed the Nutrition Toolbox, which leverages open-source databases containing metabolite composition for over ten thousand food items to convert food items into their metabolic composition to create *in silico* diets. Additionally, when used with a previously published nutrition algorithm, minimal changes to a diet can be identified to achieve desirable shifts in human and microbiome metabolism. Taken together, we believe that the Nutrition Toolbox can help to understand the effects of nutrition on human metabolism and has the potential to contribute to personalized nutrition.

**Availability and implementation:**

The Nutrition Toolbox is written in MATLAB. The code can be found at https://github.com/opencobra/cobratoolbox. A tutorial explaining the code is available in the COBRA toolbox and as view-only supplementary tutorial. Details on installing the COBRA toolbox are available at https://opencobra.github.io/cobratoolbox/stable/installation.html.

## 1 Introduction

Nutrition has long been an important area of study, particularly to maintain human health and supply nutrients (Melzer *et al.* 2021). Diet is also frequently targeted to alleviate symptoms or prevent diseases (Xiao *et al.* 2024). However, the effects of nutrients on human metabolism are not well understood (Johnson *et al.* 2020, Melzer *et al.* 2021). Studying nutrition-human metabolism is difficult due to the high variability of dietary habits and gut microbiome composition between people. The gut microbiome is a complex ecosystem that converts human and dietary metabolites. Thus, the gut microbiome from different people will consume and produce different metabolites, even if they eat the same diet (Johnson *et al.* 2020).

A method to investigate human-microbiome co-metabolism is the constraint-based reconstruction and analysis (COBRA) framework (Heirendt *et al.* 2019), in which an organism’s genome and experimental data are used to describe in a stoichiometrically accurate, mass- and charge-balanced manner the metabolic capabilities of the organism. COBRA assumes the system to be at a steady state, meaning concentrations do not change over time. The metabolic reactions can be further constrained through the addition of context-specific data, such as the uptake and excretion rates of metabolites. These additional constraints lead to the conversion from a metabolic reconstruction to a genome-scale metabolic model, which can be investigated using, for example, flux balance analysis (Orth *et al.* 2010). The COBRA framework has been applied to develop sex-specific, organ-resolved whole-body metabolic models (WBMs) that can be used to simulate human metabolism (Thiele *et al.* 2020). The WBMs can be personalized with physiological and metabolomic data. Additionally, metagenomics can be used to create community microbiome models, which can be integrated within the lumen of the large intestine in the WBMs. The high level of personalization of WBMs enables modelling nutrition-microbiome-human metabolism. However, to model the diet influence on the WBMs, the diets need to be defined on the metabolite level. Only a few metabolite-resolved diet formulations are currently available (Noronha *et al.* 2019) and thus, hamper the accurate modelling of nutrition effects on human metabolism and of nutritional interventions.

At present, several open-access food databases, including the U.S. Department of Agriculture’s FoodData Central (USDA) (U.S. Department of Agriculture Agricultural Research Service, Beltsville Human Nutrition Research Center 2024) and the National Food Institute, Technical University of Denmark (FRIDA) (National Food Institute, Technical University of Denmark 2024) database, contain metabolite-resolved information with mass measurements, e.g. grams, for a large number of food items. We define here a database food equivalent as a food item in either the USDA or FRIDA database, which could potentially be used to represent a food item consumed in the diet. Manually identifying food items in a database that match specific dietary food items, e.g. from food frequency questionnaires, is time-consuming. Additionally, converting the metabolite composition to be compatible with the WBMs requires knowledge of the virtual metabolic human (VMH) database (Noronha *et al.* 2019) and modelling conventions.

**Figure 1.**
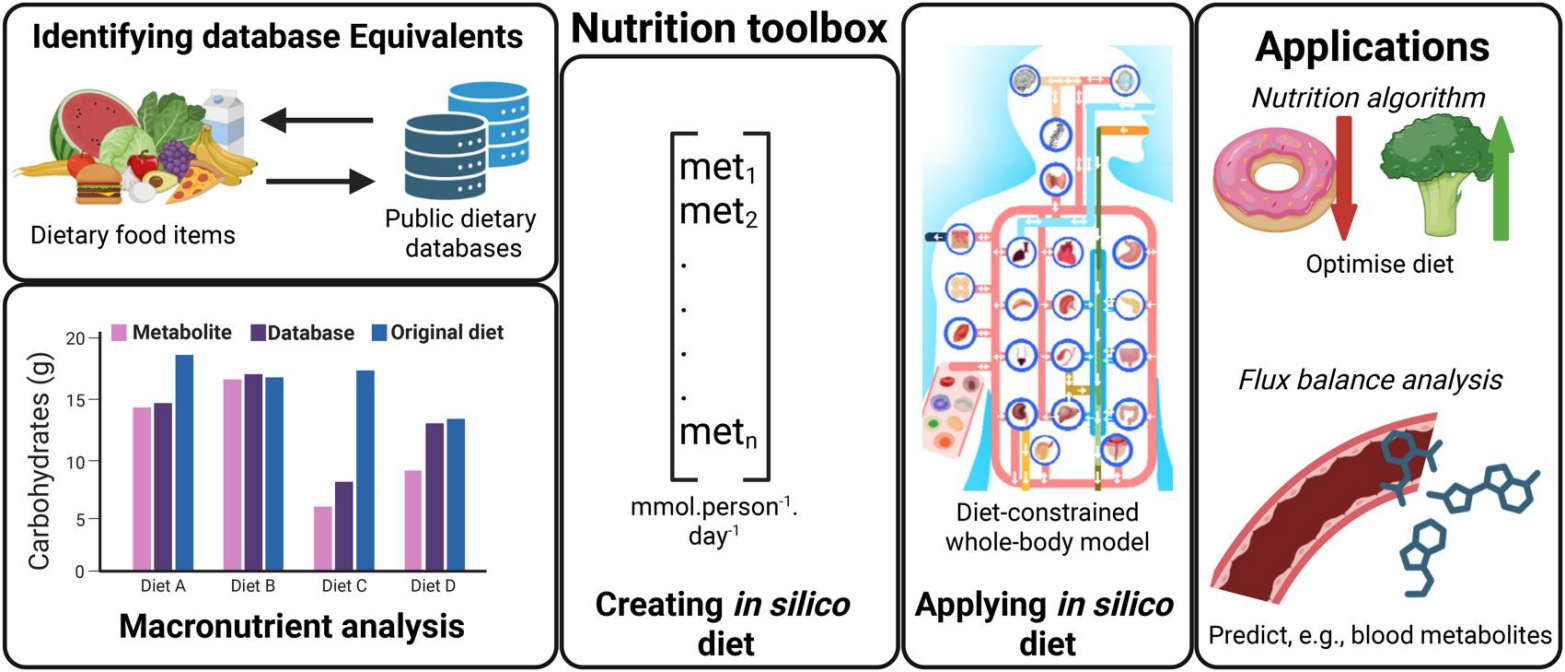
Schematic overview of the nutrition toolbox and its applications. Public dietary databases, i.e. USDA U.S. Department of Agriculture, 2024 or FRIDA National Food Institute, 2024, are used to identify database food equivalents from dietary food items based on description and macronutrient composition. The *in silico* diets are then calculated from the database food equivalents. Carbohydrate (shown), lipid, protein, sugar, and caloric contents of the *in silico* diets are compared to the original diet. To accurately compare the *in silico* diet, macronutrients as reported from the database (database in the figure) and macronutrients as calculated from measured metabolites (metabolites in the figure) are shown. The *in silico* diet can be set on the WBMs (Thiele *et al.* 2020) and be used with either flux balance analysis (Orth *et al.* 2010) to investigate human metabolism or with the nutrition algorithm (Weston and Thiele 2023) to optimize the *in silico* diet. The whole-body model figure was adapted from Thiele *et al.* (2020). Created with BioRender.

Here, we present the nutrition toolbox (Fig. 1), a MATLAB-based codebase within the COBRA Toolbox (Heirendt *et al.* 2019). The Nutrition Toolbox (i) aids in identifying database food equivalents in the USDA or FRIDA databases, (ii) can convert diets with database food equivalents into *in silico* diets, (iii) performs macronutrient analyses on *in silico* diets, (iv) sets *in silico* diets as constraints on WBMs, and (v) connects to the nutrition algorithm (Weston and Thiele 2023) to predict dietary changes. We also provide a tutorial (Supplementary Tutorial, available as supplementary data at *Bioinformatics Advances* online) explaining the various functions and a demonstration dataset in the COBRA toolbox https://opencobra.github.io/cobratoolbox/stable/tutorials/tutorial_Tutorial_nutritionToolbox.html, which are automatically downloaded during installation of the COBRA toolbox.

## 2 Methods

The nutrition toolbox is written in MATLAB (Mathworks, Inc.) and can be found in the COBRA toolbox (Heirendt *et al.* 2019). Certain parts of the nutrition toolbox perform linear optimizations, and it is recommended to use, e.g. gurobi (Gurobi Optimization, Inc.) as linear programming solver. Information on installing the COBRA toolbox, linear programming solver compatibility, and solver installation can be found at https://opencobra.github.io/cobratoolbox/stable/installation.html. The tutorial location in the cobratoolbox is cobratoolbox/tutorial/analysis/nutritionToolbox.

### 2.1 Construction of food item databases

The USDA and FRIDA databases were downloaded from https://fdc.nal.usda.gov/download-datasets and https://frida.fooddata.dk/data? lang=en, respectively. The contents of the USDA and FRIDA databases were processed for compatibility with WBMs utilizing the *createUSDAdatabase.m* and *createFridaDatabase.m* functions, respectively (available in the Nutrition Toolbox), yielding almost 14.000 food items from the USDA database and almost 1400 food items from the Frida database that could be used to formulate *in silico* diets. These functions also translate metabolites with mass measurements, i.e. variations of grams, into their respective VMH identifiers and convert these values to mmol/100g of database food equivalent.

### 2.2 Preparing dietary information to search food databases

The first step in using the Nutrition Toolbox is to prepare the dietary food items to find database food equivalents in the altered USDA/FRIDA databases. We provide a template file for users to populate with their dietary data, ensuring the correct formatting (Table 1, available as supplementary data at *Bioinformatics Advances* online). We will refer to this file throughout the manuscript as the foodDescription file. Additionally, we define a dietary food item as the food item, for which the user wants to find database food equivalents. Information required to describe dietary food items is: the dietary food item name, descriptive keywords, reference weight, and macronutrient composition (Table 1). Macronutrients are a set of nutrients that the body requires in substantial amounts. They can generally be divided into 1. carbohydrates, which can be subdivided into sugar, starch, and fibre, 2. lipids or fats, and 3. protein. The reference weight is the weight of the dietary food item for which the macronutrient composition is given, e.g. 100 g on food labels.

**Table 1.** Example of the foodDescription file that is used to find database food equivalents.^a^

OriginalFood	KeyWords	ReferenceWeight(g)	Energy (kcal)	Lipid (g)	Protein (g)	Sugars (g)
Red apple	Apple; Red	150	91.4	0.1	0.4	15.5

a Note that not all columns available are shown in this table.

Keywords should be single words separated by semicolons (;), which describe the dietary food item. A quick method for generating keywords is to separate each word from the original food name with a semicolon. The first keyword is usually the most descriptive. For example, in the case of ‘red apple’, the most descriptive term is ‘apple’, and should be the first keyword (Table 1).

The reference weight and macronutrient composition are used to assess the similarity between the dietary food items and database food equivalents. Macronutrient values can be obtained from the packaging of the dietary food item, online from distributors or general databases. Users do not have to input all available macronutrients. However, the more values are given, the more accurate and comprehensive the comparison with the database food equivalents will be. The expected units for each macronutrient are provided in the column names. When creating multiple diets, one foodDescription file can be used with all the unique dietary food items.

### 2.3 Identifying suitable database food equivalents

The function *vmhFoodFinder.m* uses the keywords and a string matching search strategy to identify potential database food equivalents. Two search strategies can be employed: sequential or cumulative. More details on the search strategies can be found in the tutorial (Supplementary Tutorial, available as supplementary data at *Bioinformatics Advances* online). Dietary food items, for which no database food equivalents are found during the keyword search, are logged in the *noDatabaseHits.txt* file. In contrast, a dietary food item may return many database food equivalents, which increases runtime. Therefore, dietary food items yielding database food equivalents exceeding a user-defined threshold (default: 50) are stored in the *tooManyDatabaseHits.xlsx* file. All database food equivalents found for dietary food items are listed and can be used for keyword refinement. For both cases, the foodDescription file will need to be updated to obtain new database food equivalents. Tips on how to handle dietary food items yielding no or too many database food equivalents, can be found in the tutorial (Supplementary Tutorial, available as supplementary data at *Bioinformatics Advances* online and in the COBRA toolbox https://opencobra.github.io/cobratoolbox/stable/tutorials/tutorial_Tutorial_nutritionToolbox.html).

The resulting database food equivalents are then passed to the *collectFoodItemInfo.m* function, where the macronutrients and metabolites for each database food equivalent are collected. The macronutrient composition extracted from the database food equivalent (database macronutrient) is subsequently compared to the user-provided macronutrient composition by calculating the Euclidean distance in the *calculateFoodScore.m*. Besides the Euclidean distance, the percentage of the measured macronutrients as metabolites is also reported. The measured metabolites are grouped into macronutrient groups and give an indication of how well the macronutrients are represented in the measured metabolite content. Database food equivalents less similar to dietary food items might be chosen if they have a higher measured metabolite representation. Carbohydrates are excluded from the macronutrient comparison due to inconsistency in definitions across databases and on food labels of dietary food items, specifically, whether fibre is included in the total carbohydrate count. Instead, individual carbohydrate components (i.e. sugars, fibre, and starch) are used, where available.

The final comparison table is saved as *fullComparison FoodItems.xlsx*. A shorter version with only the top ten hits per dietary food item is saved as *topResultsComparisonFoodItems.xlsx*.

### 2.4 Creating *in silico* diets

The most appropriate database food equivalents, chosen from the comparison files as explained in the previous section, must be linked to their consumed weights per diet. A template file is provided (Table 2, available as supplementary data at *Bioinformatics Advances* online), which requires, at a minimum, four entries (Table 2): the name of the dietary food item, the ID of the database food equivalent, the name of the database, from which the database food equivalent was selected, and the consumed weight. The file containing the consumed database food equivalents will be referred to as the equivalentsConsumed file throughout the paper. The name of the dietary food item serves as a secondary check to ensure that the correct information has been entered. The database name and ID can be found in the comparison files, from which the database food equivalents were selected. Each column following the database name represents a separate diet, where the value corresponds to the consumed weight in grams (Table 2). If a dietary food item is not consumed in a particular diet, the consumed weight should be set to zero.

**Table 2.** Example of the equivalentsConsumed file that is used to create *in silico* diets.

FoodName	databaseID	databaseOrigin	diet1 (g)	diet2 (g)
Dried date	168191	usda	50	80
Red apple	1105430	usda	100	0
Cream cheese	1215	frida	25	35

To create and analyse *in silico* diets, the function *generateInSilicoDiet.m* is used. For each consumed food in the equivalentsConsumed file, the metabolites in mmol/100g of database food equivalent are extracted, divided by 100, and multiplied by the consumed weight of the dietary food item to arrive at a unit of mmol/day/person, which can be used as constraints after summing all dietary food items. From the database food equivalents, both the database and metabolite-derived database macronutrients are visualized as bar charts. The function also calculates the fractional contribution of various macronutrients to the total energy content of the diet. If the user provides the macronutrient composition of the original diet, this info is also included in the analysis. A template is provided (Table 3, available as supplementary data at *Bioinformatics Advances* online).

To easily apply the diet to WBMs, the *setInSilicoDiet.m* function can be used. This function also uses the foodsConsumed file and offers two methods to apply the diet. The first is the conventional approach, in which the available dietary metabolites are calculated in mmol/human/day (as in *generateInSilicoDiet.m*) and directly applied to the WBMs. Alternatively, database food equivalents themselves can be used as constraints. As WBMs are not natively able to ’consume’ foods, additional reactions must be added to the WBMs. Two types of reactions are included for each database food equivalent in the selected database(s). The first is an exchange reaction, allowing for ’consumption’ of the database food equivalent, defined as:


(1) foodItem[f]←

where [f] is a new compartment denoting the food source. The second is a breakdown reaction, representing the conversion of the database food equivalents into its constituent metabolites and macronutrients. These reactions are defined as:


(2) $$\text{foodItem}[f]\to {\text{met}}_{1}[d]+\ldots {+\text{met}}_{n}[d]$$

where *n* is the total number of product metabolites and [d] the dietary compartment. Note that macronutrients (e.g. energy, lipids) are treated as metabolites in this approach for modelling purposes, but do not interact with the metabolic network of the WBMs and are not broken down into specific molecules. They are only used to calculate the macronutrient composition of the ’ingested’ diet in the WBMs and to enable control over dietary composition when working with the nutrition algorithm. Finally, exchange reactions are added for these newly defined macronutrients to ensure mass balance within the WBM and to allow the macronutrients to be removed from the WBM, now called food item-constrained WBMs, formulated as:


(3) macro[d]→

It is recommended to use the food item-constrained WBMs primarily when the objective is to adjust the diet itself. For standard analyses of diet impact on WBMs, the use of metabolite-constrained WBMs is advised, as the inclusion of numerous exchange and breakdown reactions can significantly increase model size and complexity. Lastly, the function includes a feasibility check for the resulting WBMs. If essential metabolites are missing, the diet is automatically adjusted to restore model feasibility. Feasibility for WBMs is defined as the WBM capability to satisfy the whole-body maintenance objective. For community microbiome WBMs, the production of community microbiome biomass is added as an additional feasibility check. More information on ensuring feasibility can be found in the tutorial (Supplementary Tutorial, available as supplementary data at *Bioinformatics Advances* online).

### 2.5 Integration with the nutrition algorithm

To further leverage the information contained within food databases, the previously published nutrition algorithm (Weston and Thiele 2023) was adapted to work with the WBMs and the nutrition toolbox. The nutrition algorithm can use metabolite-constrained and food item-constrained WBMs to determine the minimal dietary change required to achieve the greatest increase or decrease in specific metabolic fluxes of interest (e.g. decrease of blood cholesterol levels). Dietary modifications can be calculated at the metabolite or database food equivalent level. Applications of the nutrition algorithm at the metabolite level may be most helpful for the identification of novel food supplements, while applications at the database food equivalent level could be useful for creating a personalized diet or identifying optimal diets to target various diseases.

When utilizing food-item optimization, constraints can be specified to limit how much specific macronutrients vary. For example, a user can request the minimal changes to a given diet to maximally increase dopamine storage in the brain while maintaining lipid intake and allowing a maximum addition or removal of 200 grams of food. Additionally, the user can specify the caloric range (in kcal) of the consumed diet. These additional constraints on macronutrients enable precise control over dietary modifications compared to those produced without macronutrient constraints.

### 2.6 Limitations

WBMs do not take into account enzyme kinetics or bioavailability of nutrients, which is important to consider when interpreting the simulation results, as the modelled intake of nutrients through the gastrointestinal tract might not accurately reflect *in vivo* uptake. Secondly, the predictive capability of an *in silico* diet generated by the nutrition toolbox is limited by the number of metabolites measured for a database food equivalent, which can vary between databases and between food equivalents in the same database. Any predicted changes in metabolism or diet, through the nutrition algorithm, are thus hypotheses and need to be validated through literature research or experiments.

Although the Nutrition Toolbox was designed for the WBMs, only *setInSilicoDiet.m* is specifically formulated for the WBMs. The generated *in silico* diets can be applied to any metabolic model, given that they have the VMH namespace. Alternatively, metabolite IDs need to be renamed to fit the namespace of the chosen metabolic model.

## 3 Conclusion

The Nutrition Toolbox provides a workflow to integrate publicly available food databases with the power of WBMs to enable researchers in the fields of metabolic modelling and nutrition to investigate the effects of diet composition on human metabolism. Potential research questions that could be investigated with the Nutrition Toolbox could be how the metabolic activity of an individual’s microbiomes changes with a specific diet, dietary intervention, or the effect of personalized nutrition on disease metabolism modulation. In conjunction with the nutrition algorithm, minimal dietary alterations can be calculated to achieve a targeted change in flux through specific metabolic reactions, which can be used to suggest novel prebiotics or dietary solutions to improve human health. The Nutrition Toolbox and the nutrition algorithm represent a step towards the efficient and user-friendly metabolic understanding of nutrition and the development of personalized nutrition strategies.

## Supplementary Material

vbaf325_Supplementary_Data

## Data Availability

The code can be found at https://github.com/opencobra/cobratoolbox. A tutorial explaining the code is available in the COBRA toolbox and as view-only supplementary tutorial. Details on installing the COBRA toolbox are available at https://opencobra.github.io/cobratoolbox/stable/installation.html.
