# Supplementary material for: The nutrition toolbox permits in silico generation, analysis, and optimization of personalized diets through metabolic modelling
Source: Bioinform Adv. 2026 Jan 8;6(1):vbaf325. doi: 10.1093/bioadv/vbaf325 (PMC12820401; doi:10.1093/bioadv/vbaf325)
Supplement: vbaf325_Supplementary_Data [file vbaf325_supplementary_data.zip › Tutorial_nutritionToolbox.html]

The nutrition toolbox to generate in silico diets 

# The nutrition toolbox to generate in silico diets

## Author(s): Bram Nap (University of Galway, Ireland)

## Reviewer(s):

Cyrille C. Thinnes (University of Galway, Ireland)

## INTRODUCTION

This is a tutorial to show how to use the nutrition toolbox to a) find database food equivalents in the USDA [1] or FRIDA [2] databases, b) use the database food equivalents to create in silico diets, c) how to set in silico diet constraints on whole-body metabolic models[3], and d) how to connect to the nutrition algorithm[4] to predict dietary changes. A database food equivalent here means a food item in either the USDA or FRIDA database.

## MATERIALS

- MATLAB.
- COBRA toolbox [5]. For instruction on installing the COBRA toolbox see https://opencobra.github.io/cobratoolbox/stable/installation.html
- Food items with corresponding macronutrient information (see template file in the nutrition toolbox paper).
- Diet file with consumed dietary items per diet (see template file in the nutrition toolbox paper).
- Total macrocomposition of diets (optional) (see template file in the nutrition toolbox paper).
- Industrial solver. IBM cplex, MOSEK, gurobi - see https://opencobra.github.io/cobratoolbox/stable/installation.html# for solver installation and compatibilities.

## PROCEDURE

#### Step 1. Prepare your input files

Before we can start using the codebase we need to properly format and fill in the input files.

The nutrition toolbox uses three input files, one of which is optional. The first input file contains the macronutrient composition and associated consumed weight of each food item, referred to as the foodDescription file (you may also use the default values found on the packaging, in which case the weight consumed is 100g or equivalent depending on the information available). An example can be found in the file demoFoodDescription.xlsx. We also provide a partially completed file with the macronutrient information already filled in (demoFoodDescriptionEmpty.xlsx), so you can test filling in keywords. The columns in the files are:

- OriginalFoodName - the original name of the food item. This would be the name you, the user, would refer to the food item. It has no influence on the results.
- KeyWords - semicolon (;) seperated list of words that describe the food item. Ensure that the first keyword is the most descriptive or important as the code uses that in weighting potential database food equivalents. E.g., for the food item "red apple", the most descriptive word is apple, so we shall put this as the initial keyword. A method to quickly generate keywords is to separate each word in the original food name with a semicolon.
- toExclude - semicolon (;) seperated list of key words that exclude results from the database food equivalent search.
- databaseID - semicolon (;) seperated list of database IDs already found through previous iterations of running the algorithm.
- databaseName - semicolon (;) seperated list of the name of the database corresponinding to the IDs filled in the databaseID column. This is either usda or frida and nothing else.
- ReferenceWeight(g) - The reference amount of food that matches the reported marconutrient values. This would typically be 100g for most food labels. Always given in grams
- Alcohol(g) - Amount of alcohol reported on the food label.
- TotalEnergy(kcal) - Amount of energy reported on the food label. IMPORTANT given in KCAL.
- TotalFibre(g) - Amount of fibre reported on the food label.
- TotalLipid(g) - Amount of lipids reported on the food label, including both saturated and unsaturated fatty acids.
- TotalProtein(g) - Amount of protein reported on the food label.
- TotalStarch(g) - Usually not given on normal food labels, yet included here in case this information is available to the user, e.g., through consulting other sources.
- TotalSugar(g) - Amount of sugar reported on the food label. Usually it will say Carbohydrates -> of which sugars.
- TotalWater(g) - Amount of water in the food, usually not given on normal food labels.

Notably, total carbohydrates are excluded from the comparison due to inconsistency in definitions across databases and on food labels of dietary food items, specifically whether fibre is included in the total carbohydrate count. Instead, individual carbohydrate components (i.e., sugars, fibre, and starch) are used where available.

If information for a macronutrient column is not available, leave the cell blank.

If the label of your food is given as a volume instead of weight (e.g., /100mL) you will need to convert the volume into weight, e.g., by measuring how many grams the 100mL correspond to, or converting the volume into to grams by using the food item density (if available).

If a food item is consumed in different quantaties across different diets, you only have to enter it once foodDescriptions file. IMPORTANT is that reported macronutrients correspond to the reference weight (includes water weight)

The reference weights are used to to adjust the macronutrients from database food equivalents to the same weight consumption as the macronutrients for database food equivalents are all given per 100g of food consumed.

Users should double-check that the reported macronutrients correspond the reference weight of the food item. I.e., make sure that if the reference weight is 80g, not the standard 100g of food on food labels is input in the various macronutrient columns.

The second input file contains the consumed weight of food items per diet which will be used to create in silico diets, referred to here as the foodsConsumed file. In the foodsConsumed file we will fill in the database food equivalents found with the foodDescription file. If a food item is not consumed in a diet, set the weight consumed to 0. A worked example can be found in demoFoodConsumed.xlsx, an example without the database food equivalents can be found in demoFoodConsumedEmpty.xlsx. The following columns can be filled in:

- originalName - The original food name. This is usefull to double check that the consumed weights per diet are correct and the correct database food equivalents are put in place.
- foodName - The name of the database food equivalent, serves as a control to make sure it matches with the original food.
- databaseID - The ID of the database food equivalent - this will be given in the output of step 2 when we find and compare database food equivalents.
- databaseUsed - The name of the database where the database food equivalent is found - this will be given in the output of step 2 when we find and compare database food equivalents.

The third input file contains the macronutrient composition of the full diets and can found in demoOriginalMacroDiet. Each column is a diet with the rows being the relevant macronutrients.

Then we initialise the COBRA toolbox and set our industrial solver

% Initialise the toolbox

initCobraToolbox();

\_\_\_\_\_ \_\_\_\_\_ \_\_\_\_\_ \_\_\_\_\_ \_\_\_\_\_ |
/ \_\_\_| / \_ \ | \_ \ | \_ \ / \_\_\_ \ | COnstraint-Based Reconstruction and Analysis
| | | | | | | |\_| | | |\_| | | |\_\_\_| | | The COBRA Toolbox - 2025
| | | | | | | \_ { | \_ / | \_\_\_ | |
| |\_\_\_ | |\_| | | |\_| | | | \ \ | | | | | Documentation:
\\_\_\_\_\_| \\_\_\_\_\_/ |\_\_\_\_\_/ |\_| \\_\ |\_| |\_| | http://opencobra.github.io/cobratoolbox
|
> Checking if git is installed ... Done (version: 2.37.3).
> Checking if the repository is tracked using git ... Done.
> Checking if curl is installed ... Done.
> Checking if remote can be reached ... Done.
> Initializing and updating submodules (this may take a while)... Done.
> Adding all the files of The COBRA Toolbox ... Done.
> Define CB map output... set to svg.
> TranslateSBML is installed and working properly.
> Configuring solver environment variables ...
- [---\*] ILOG\_CPLEX\_PATH: C:\Program Files\ibm\ILOG\CPLEX\_Studio1210\cplex\matlab\x64\_win64
- [\*---] GUROBI\_PATH: C:\gurobi1201\win64\matlab
- [\*---] TOMLAB\_PATH: --> set this path manually after installing the solver ( see instructions )
- [\*---] MOSEK\_PATH: --> set this path manually after installing the solver ( see instructions )
Done.
> Checking available solvers and solver interfaces ...Could not find installation of dqqMinos, so it cannot be tested
Could not find installation of matlab, so it cannot be tested
Could not find installation of mosek, so it cannot be tested
Could not find installation of quadMinos, so it cannot be tested
Could not find installation of tomlab\_snopt, so it cannot be tested
Done.
> Setting default solvers ...Could not find installation of mosek, so it cannot be tested
Could not find installation of mosek, so it cannot be tested
Could not find installation of matlab, so it cannot be tested
Done.
> Saving the MATLAB path ... Done.
- The MATLAB path was saved in the default location.
> Summary of available solvers and solver interfaces
Support LP MILP QP MIQP NLP EP CLP
------------------------------------------------------------------------------
dqqMinos active 0 - 0 - - - -
glpk active 1 1 - - - - -
gurobi active 1 1 1 1 - - -
lp\_solve legacy 1 - - - - - -
matlab active 0 - - - 0 - -
mosek active 0 - 0 - - 0 0
pdco active 1 - 1 - - 1 -
qpng passive - - 1 - - - -
quadMinos active 0 - - - - - -
tomlab\_snopt passive - - - - 0 - -
------------------------------------------------------------------------------
Total - 4 2 3 1 0 1 0
+ Legend: - = not applicable, 0 = solver not compatible or not installed, 1 = solver installed.
> You can solve LP problems using: 'gurobi' - 'glpk' - 'pdco'
> You can solve MILP problems using: 'gurobi' - 'glpk'
> You can solve QP problems using: 'gurobi' - 'pdco'
> You can solve MIQP problems using: 'gurobi'
> You can solve NLP problems using:
> You can solve EP problems using: 'pdco'
> You can solve CLP problems using:
> Checking for available updates ...
> There are 132 new commit(s) on <master> and 28 new commit(s) on <develop> [b75356 @ develop]
> You can update The COBRA Toolbox by running updateCobraToolbox() (from within MATLAB).
removing: C:\Users\MSPG\Documents\cobratoolbox\src\analysis\thermo\componentContribution\new
removing: C:\Users\MSPG\Documents\cobratoolbox\src\analysis\thermo\groupContribution\new
removing: C:\Users\MSPG\Documents\cobratoolbox\src\analysis\thermo\inchi\new
removing: C:\Users\MSPG\Documents\cobratoolbox\src\analysis\thermo\molFiles\new
removing: C:\Users\MSPG\Documents\cobratoolbox\src\analysis\thermo\protons\new
removing: C:\Users\MSPG\Documents\cobratoolbox\src\analysis\thermo\trainingModel\new

% Change the solver, can be gurobi, ibm\_cplex, tomlab\_cplex, or mosek

changeCobraSolver('gurobi');

> changeCobraSolver: Gurobi interface added to MATLAB path.

Shortly on how the USDA and FRIDA databases were created. The functions createUSDAdatabase and createFridaDatabase are used to convert food items into VMH readable format but are not discussed here in this tutorial. The nutrients per food item are exctracted and converted to mmol. To obtain mmol/100g database food equivalent for the metabolite content of the databases, we have transformed the original database measurements. The databases contain measured metabolite weights in the format of g (or form thereof) /100g database food equivalent. We have converted them into g/100g of food item for easy manipulation and then divided them by the molecular weight (g/mol) of a metabolite to arrive at mol/100g. Finally, multiplication by a 1000 transforms the data into mmol/100g of database food equivalent.

#### Step 2: Finding database food equivalents.

The first step in making an in silico diet is to find food equivalents in databases that have metabolite compositions measured. Here we use both the the U.S. Department of Agriculture, FoodData Central (USDA) database and the National Food Institute, Technical University of Denmark FRIDA (FRIDA) database. The function we use to find database food equivalents is vmhFoodFinder.m and is available in the COBRA toolbox. The function takes the following inputs:

- templateFilePath Required - The full file path to where the input file is stored. IMPORTANT! remember to include a file extension. The demo file for this step is the demoFoodDescription.xlsx file.
- searchType Optional - Method of searching keywords in the food database. Either sequential or cumulative. Explanation about the difference is given below.
- addStarch Optional - Boolean, indicates if additional starch should be added based on the reported macronutrients. It will add on additional starch based on the equation starch = total carbohydrates - fibre - total sugars, as starch is often not measured in the USDA database. Defaults to false.
- databaseType Optional - Character, which database should be used either 'usda' for USDA FoodData database or 'frida' for the Danish food institute database. Can be 'mixed' if both databases should be used. Defaults to mixed.
- maxItems Optional - Numeric, value indicating the maximum amount of database food equivalents to be analysed for macronutrients. Defaults to 50.
- outputDir Optional - Path to where the output file will be stored. Defaults to [pwd filesep 'NT\_Result']
- foodSources2Use Optional - Cell of strings, dictates which food sources from the USDA database will be used to find food items. Defaults to {'sr\_legacy\_food'; 'foundation\_food'; 'survey\_fndds\_food'};

There are two search methods employed to find potential database food equivalents: sequential and cumulative

In the sequential search method, the first keyword is used to identify database food equivalents containing that keyword, resulting in sub-selection 1. The second keyword is then used to filter sub-selection 1, retaining only those entries that contain the second keyword, thereby producing sub-selection 2. This process continues until all keywords have been used. If the final sub-selection is empty, the results from the second to last sub-selection are used, if applicable.

In the cumulative search method, each keyword independently generates a sub-selection of database food equivalents that contain the respective keyword. All resulting database food equivalents are then tallied. Database food equivalents with a total count of total number of keywords -1 or more are returned. If no database food equivalents reach that threshold, the results for total number of keywords -2 matches are returned.

First we will define some of the input arguments

% Path to the input file, change to where the file is on your computer

templateFilePath = 'C:\nutritionToolbox\demoFoodDescription.xlsx';

% Path to where we want our results stored so we can easily find them,

% change to an existing path on your computer

outputDir = 'C:\nutritionToolbox\results';

If we want to adjust more input arguments we can define them by pressing enter to add additional lines after line 5, and copy and pasting the input argument we want to alter. Add an equal sign (=) and the new value and end with a semicolon. E.g., if you want to decrease the number of items to be compared, you would put maxItems = 30; underneath line 5.

To call the function (Note for the demo it will take likely between 10 to 20 minutes for the initial run):

vmhFoodFinder(templateFilePath, 'outputDir', outputDir);

Warning: The following USDA food ID does not seem to have a KCAL assocatiated with it. Please take note:321505

Warning: The following USDA food ID does not seem to have a KCAL assocatiated with it. Please take note:2685568

Warning: The following USDA food ID does not seem to have a KCAL assocatiated with it. Please take note:321505

Warning: The following USDA food ID does not seem to have a KCAL assocatiated with it. Please take note:2685568

If you defined multiple input arguments, add them after outputdir as 'name as defined in the list above' and the variable name where the new value is stored or the value directly. E.g., if you want to adjust maxItems, you can either set ,'maxItems', maxItems if you defined maxItems before OR set ,'maxItems', 30 behind outputdir. The resulting input would look like vmhFoodFinder(templateFilePath, 'outputDir', outputDir, 'maxItems, maxItems); OR vmhFoodFinder(templateFilePath, 'outputDir', outputDir, maxItems, 30);

The outputs will be stored in a folder called NT\_results in the defined directory set in the outputDir variable. If left empty, it will be made in your MATLAB current working directory.

Four output files will be stored in the NT\_results folder

- fullComparisonFoodItems.xlsx - The file contains the full comparison for each food item. Food items that had no, or too many, database food equivalents have also been indicated. The file can be used to select database food equivalents to create in silico diets with.
- tooManyDatabaseHits.xlsx - This file contains all the food items that had more than the maximum allowed database food equivalents to be compared (set in the maxItems input variable). The columns contain the food items and the rows contain every database food equivalent found for that food item. The file can be used to refine the key words used to describe food items, or to populate the toExclude column in the input file.
- noDatabaseHits.txt - A list of food items for which no database food equivalents were found. For these items, check the spelling of key words, food equivalents names, or reduce the amount of key words. If that does not help, try to find a database food equivalent on VMH.life.
- topResultsComparisonFoodItems.xlsx - Similar to fullComparisonFoodItems.xlsx, containing only the top 10 results for a more concise overview of the results.

Either the fullComparisonFoodItemsn or topResutlsComparisonFoodItems can be used to select database food equivalents. The code compares the macronutrients provided by the user for each food item and compares them to the given/measured macronutrients from the database food equivalents (so-called label macronutrients) and then uses Euclidean distance to evaluate how similar the two are. The Euclidean distance is given in the SimilarityScore column of these two files. Besides the Euclidean distance, the percentage of the macronutrients measured as metabolites is also reported. The measured metabolites are grouped into macronutrient groups and give an indication of how well the macronutrients are represented in measured metabolite content. Database food equivalents less similar to dietary food items may be chosen if they have a higher measured metabolite representation in their macronutrients. By choosing the database food equivalents that may be less similar to the dietary food item but with a higher measured metabolite representation, it increases the metabolic content of the in silico diets and give more overall information as opposed to choosing database food equivalents with lower metabolite representation that might be more similar to the dietary food item.

For example, if we were to compare two database food equivalents: "Dates, medjool" and "Date", both from the USDA database, the reported macronutrient values are near identical. However, "Dates, medjool" has higher percentages of macronutrients covered by measured metabolites than "Date", making it a better choice due to its greater metabolite coverage.

It is very likely that you will need to perform 2 or 3 runs with updated keywords to obtain suitable database food equivalents for each dietary food item. To reduce time, it is recommended to fill in the column databaseID and databaseName for the dietary food items for which appropriate database food equivalents have been identified. To improve keyword performance in subsequent runs, users may consider changing the number of keywords, exploring alternative spellings, or naming conventions. Additionally, looking at the tooManyDatabaseHits may help finding keywords for the toExclude column. Alternatively, you can increase the max items compared, set by putting ,'maxItems', x in the function call (where x is the new number of max items compared).

#### Step 3: Generating in silico diets

With step 1 and step 2 performed, we can now prepare the second input file, referred to as "demoCreateDiet" here. In step 1, we already filled in the original food items and the consumed weight per diet in the "demoCreateDiet" file. Now we can fill in the database id and database name for each dietary food iem. The two fields can be directly copied from fullComparisonFoodItemsn or topResutlsComparisonFoodItems where the relevant database food equivalents were chosen. Once completed, we can use the function generateInSilicoDiet.m to create our in silico diets and do some quick analyses. The function takes the following inputs:

- toCreateDiet Required: Path to the file with the diets that need to be created. consist of column "originalName" where the orignal food names are set. "databaseID" the ID of the fooditem in their database. "databaseUsed" which database was used to find that database food equivalent. Each column after is a diet where the values in gram show the food items consumed for each diet. The demo file for this is called 'demoCreateDiet.xlsx'.
- outputDir Optional: Path to the directory where the results should be stored. Defaults to current working directory.
- originalDietMacros Optional: Path to the file with the total macronutrients for the original diets. Should contain the rows lipids, carbohydrate, protein and energy. Defaults to an empty string, i.e., not provided. Template how the file should look like can be found with the name 'demoOriginalMacroDiet.xlsx'.
- analyseMacros Optional: Boolean, indicates if analysis on macronutrients should be performed. Defaults to true.
- addStarch Optional: Boolean, indicates if additional starch should be added based on the reported macronutrients. It will add on additional starch based on the equation starch = total carbohydrates - fibre - total sugars, as starch is often not measured in the USDA database. Defaults to false.

First let us define our variables

% The path to your diet file. The demo is called demoCreateDiet.xlsx.

% Change to the path where the file is on your computer

toCreateDiet = 'C:nutritionToolbox\demoCreateDiet.xlsx';

% We initialised outputDir in step 2 already. If you want to store it in a

% different directory, remove the % from the code below and put in the

% desired location.

% outputDir = '';

% If you have the macronutrients for the original diets fill the

% demoOriginalMacroDiet.xlsx file and give the path here. If you dont have

% it leave it as is. Change to the path where the file is on your computer.

originalDietMacros = 'C:\nutritionToolbox\demoOriginalMacroDiet.xlsx';

% Put to false if you do not want analysis on the macronutrients

analyseMacros = true;

% Set to true if you want to add on additional starch for USDA based

% database food equivalents

addStarch = false;

% Now we can the function

[dietFlux] = generateInSilicoDiet(toCreateDiet, 'outputDir', outputDir, ...

'originalDietMacros', originalDietMacros, ...

'analyseMacros', analyseMacros, 'addStarch', addStarch);

The figure generated by the code will show you how well various macro-nutrients match the original diet. In blue are the macronutrients calculated from measured metabolites, in orange the macronutrients as measured from the database food equivalents and in yellow the reported macronutrients from the original diet. From the demo we can see that the database food equivalents and the original diet match well in macronutrient composition, with only a slight overestimation of lipid content. We see that the macronutrients calculated from measured metabolites (blue bar) is lower. This is because the metabolite content for every database food equivalent is not perfectly represented and some metabolites might be missed or not measured. There are also measurement errors that could play a small role in the deviations we see.

Caloric values for database equivalents are extracted from the database. Caloric values for metabolites are assigned based on which macronutrient category a metabolite falls into. lipids are assigned 9 kcal/g macronutrient, protein and sugars 4 kcal/g macronutrient, and alcohol 7 kcal/g macronutrient[6]. Metabolites falling outside these categories are assigned a caloric value of 0 kcal/g macronutrient.

We can look at the first 10 rows of dietFlux and see that indeed the dietary metabolites are returend for the diet that we created. It is normal that not all dietary metabolites have an asocciated flux value as not every metabolite is a) measured or b) found in every food item / database. The flux values are given in mmol/human/day

dietFlux(1:10,:)

ans = 10×2 table

|  | VMHID | Diet1 |
| --- | --- | --- |
| 1 | 'Diet\_EX\_25hvitd3[d]' | "0" |
| 2 | 'Diet\_EX\_4hpro\_LT[d]' | "0" |
| 3 | 'Diet\_EX\_M01582[d]' | "0" |
| 4 | 'Diet\_EX\_M02053[d]' | "0" |
| 5 | 'Diet\_EX\_M03045[d]' | "0" |
| 6 | 'Diet\_EX\_M03051[d]' | "0" |
| 7 | 'Diet\_EX\_ala\_L[d]' | "18.62491657" |
| 8 | 'Diet\_EX\_arach[d]' | "0.5792149427" |
| 9 | 'Diet\_EX\_arachd[d]' | "0" |
| 10 | 'Diet\_EX\_arg\_L[d]' | "11.66127926" |

All results are stored in the inSilicoDiet folder created in the output directory defined at the start with the following files:

- fluxDiets.csv - This file contains the flux values for all the dietary components for each diet.
- macrosCalculatedFromMetabolites.csv - The macronutrients as calculated from the measured metabolites for each diet.
- macrosAsReported.csv - The macronutrients as reported for all the food items that make up each diet.
- macroComparison.png - The figure made output by MATLAB, showing the comparison for each diet between the various macronutrients and how well the metabolite macronutrients align with the reported/label macronutrients and, if provided, the original diet macronutrients.
- energyFractionPerMacro.csv - For the lipids, carbohydrates and the proteins - the fraction of their contribution to the total energy of the diet. Calculated for the metabolite macronutrients, reported/label macronutrients and, if provided, the original macronutrients.

These files can be used to gain a deeper understanding of diet. The total metabolite macronutrients (the macronutrients calculated from measured metabolites) compared to the actual measured macronutrients of the in silico diets give an understanding on how well the overall macronutrients are represented by measured metabolites. Comparing the measured macronutrients of the in silico diets and the total macronutrients from the original diet will give an insight as to how well the in silico diet matches the original diet.

The file fluxDiets.csv or the output fluxDiets can be used to constrain WBMs with. However we can also use the function setInSilicoDiet.m to easily set the diet with all the correct checks and save them. The function can also check if the WBMs are feasible on the given diet and add on required micronutrients where required. Besides setting the diet altering the dietary exchange reactions, we can also have the WBMs "consume" the food items themselves. In order to do that the toolbox adds in novel reactions to allow for food intake and food breakdown. The food intake is straightforward, as it will be an exchange reaction of the food item (now treated as a metabolite) as in the novel food ([f]) compartment

foodItem[f] <-

This allows for food items to enter the WBMs. Then we to breakdown the food item into its metabolite components.

foodItem[f] -> met1[d] + ... + metx[d]

Where x is the number of metabolites measured for the food item. We also incorporate macronutrients such as energy and total lipids so we can track them in the diet. The macronutrients here are treated as metabolites. In order to comply with mass balance rules, we need to remove the macronutrients we are treating as metabolites from the system. We do this by setting another exchange reaction.

macronutrient[d] ->

With these new reactions in place the WBMs can consume and breakdown food items into their metabolite components and we can track the total amount of various macronutrients in the diet. Important to note is that the food exchange, breakdown, and macronutrient exchange reactions are in g/person/day (kcal/person/day for energy consumption) instead of the usual (mmol/person/day). You can choose either or both options to constrain your WBMs with in setInSilicoDiet.m but we advise to only use the food item-constrained WBMs in conjuction with the nutrition algorithm or to do simulation on diet composition. The additional food item reaction can make the vastly increase the reaction content of the WBMs (by over 30.000 reactions) and make the WBM overly complex for questions that do not involve the optimisation of the actual food items. To study the effect of diet on the various reactions in the human body - we suggest to just use metabolite-constrained WBMs. If no metadata is provided, the function will create both a female and a male WBM for each diet. If metadata is provided the diets will be matched to the corresponding sex-specific WBMs. You also have the possibility to give your own WBMs if you do not want to use the standard Harvey/Harvetta (e.g., community microbiome WBMs). If you have sample specific WBMs, ensure that the sample ID is in the file name of your WBM flanked by underscores. E.g., WBM\_sample1\_female.mat. If you have altered WBMs that are not sample specific, i.e., one altered male WBM and one altered female WBM, ensure the filename include "\_male" and "\_female", respectrively. I.e., WBM\_altered\_male.mat or WBM\_altered\_female.mat.

The setInSilicoDiet.m function takes the following inputs:

- dietToSet Required - Path to the file with the diets that need to be created. Consists of column "originalName" where the orignal food names are set. "databaseID" the ID of the fooditem in a database. "databaseUsed" which database was used to find that database food equivalent. Each column after is a diet where the values in gram show the food items consumed for each diet. The demo file for this is called 'demoCreateDiet.xlsx'.
- metadataPath Optional - Path to the metadata file, should contain the columns ID, sex and diet. Defaults to an empty string, i.e., not provided.
- outputDir Optional - Path to the directory where the results should be stored. Defaults to an empty string, i.e., not provided.
- constrainFoodWBM Optional - Boolean, indicates if the code should alter WBMs to 'consume' food items and set the diet as food items instead of dietary fluxes. Defaults to false.
- constrainFluxWBM Optional - Boolean, indicates if WBM should be constrained with the dietary exchange fluxes. Defaults to true.
- checkFeasibility Optional - Boolean, indicates if the newly constrained WBMs should be tested for feasibility. If they are not feasible, dietary exchange reactions are added to try and make them feasible. Defaults to true.
- wbmVersion Optional - If a specific version of the WBMs should be used. Do not start with \_. Defaults to an empty string, i.e., not provided.
- pathToWBMs Optional - If non-default WBMs are to be used. Default WBMs are unchanged Harvetta/Harvey. Path to where the WBMs are stored that need to be constrained. If you have sample specific WBMs, ensure that the sample ID is in the file name of your WBM flanked by underscores. E.g., WBM\_sample1\_female.mat. If you have altered WBMs that are not sample specific, i.e., one altered male WBM and one altered female WBM, ensure the filename include "\_male" and "\_female", respectrively. I.e., WBM\_altered\_male.mat or WBM\_altered\_female.mat.

Let us now define the variables. Setting more diets and setting diets on more models will increase the time of this step. For the demo this step should take ~5 minutes

% This is the same as toCreateDiet set previously. Change to where the file

% is on your computer.

dietToSet = 'C:\nutritionToolbox\demoCreateDiet.xlsx';

% The path to the metadata file if available. Otherwise standard female and

% male WBMs will be constrained for each diet.

metadataPath = '';

% We initialised outputDir in step 2 already. If you want to store it in a

% different directory, remove the % from the code below and put in the

% desired location.

% outputDir = '';

% Change to false if you do not want the WBMs to be constrained with food items

constrainFoodWBM = true;

% Change to false if you want to WBMs to be constrained with dietary flux

% values

constrainFluxWBM = true;

% Change to false if you do not want to check if WBMs are feasible on the

% constrained diets.

checkFeasibility = true;

% Change if you want to use a specific version of the WBMs, otherwise it

% will take the latest version available. If you want to use a specific

% version do not start with \_

% The results in this tutorial were made with wbmVersion 1\_03d in the COBRA

% toolbox and for testing to see if the same results are achieved, we

% recommend using this version.

wbmVersion = '1\_03d';

% Change if non-default WBMs are to be used. Path to where the WBMs are stored

% that need to be constrained. If general models, use \_female or \_male

% to indicate the sex of the generic model. Otherwise ensure that the

% sample ID is flanked by underscores (e.g., \_sample1\_).

pathToWBMs = '';

% Set all the inputs and run the code

setInSilicoDiet(dietToSet, 'metadataPath', metadataPath, 'outputDir', outputDir, ...

'constrainFoodWBM', constrainFoodWBM, 'constrainFluxWBM', constrainFluxWBM, ...

'checkFeasibility', checkFeasibility, 'wbmVersion', wbmVersion, ...

'pathToWBMs', pathToWBMs);

Setting dietary flux vector on WBMs for Diet1
Setting food items on WBM for Diet1
> changeCobraSolver: Gurobi interface added to MATLAB path.
EnsureGrowthOnDiet -- STEP 1: Test which models are feasible on the given diet

Warning: Reaction Excretion\_EX\_microbiota\_LI\_biomass[fe] not in model

Warning: Reaction Excretion\_EX\_microbiota\_LI\_biomass[fe] not in model

Model Diet1\_fWBM\_female is feasible on the given diet

Warning: Reaction Excretion\_EX\_microbiota\_LI\_biomass[fe] not in model

Warning: Reaction Excretion\_EX\_microbiota\_LI\_biomass[fe] not in model

Model Diet1\_fWBM\_male is feasible on the given diet
All models are feasible on the diet. No dietary alterations are needed.
> changeCobraSolver: Gurobi interface added to MATLAB path.
EnsureGrowthOnDiet -- STEP 1: Test which models are feasible on the given diet

Warning: Reaction Excretion\_EX\_microbiota\_LI\_biomass[fe] not in model

Warning: Reaction Excretion\_EX\_microbiota\_LI\_biomass[fe] not in model

Model Diet1\_WBM\_female is feasible on the given diet

Warning: Reaction Excretion\_EX\_microbiota\_LI\_biomass[fe] not in model

Warning: Reaction Excretion\_EX\_microbiota\_LI\_biomass[fe] not in model

Model Diet1\_WBM\_male is feasible on the given diet
All models are feasible on the diet. No dietary alterations are needed.

New directories will be created in the chosen output directory. One is called fluxDietWBMs, if you chose to constrain WBMs with dietary flux reactions, and one called foodDietWBMs, if you chose to constrain with food items. In each folder, the various diet-constrained WBMs are stored.

You can choose the check if the WBMs are feasible on the new in silico diets. Feasibilty means that enough metabolites can are consumed and created to satisfy the whole-body maintenance reaction (usually set to [1,1] /day/person) Reaction ID "Whole\_body\_biomass\_reaction" and if applicable to produce community microbiome biomass (usually set to [1,1] /day/person). If feasibilities are checked Excel file named dietGrowthStats.xlsx is created. The dietGrowthStats file contains WBM feasibility on a unconstrained diet (all dietary metabolites available) and on the constrained diet, as well as the feasibility on the constrained diet with adjustments made try and make infeasibile WBMs feasible.

The attempt to make make infeasbile WBMs feasible again, the function ensureWBMfeasibility.m is used. It first checks if the WBM is feasible on the given diet. WBMs that are infeasible on the given diet, are then given an unconstrained diet where all dietary exchange reactions have their lower bound set to -1000000. This is done to check if dietary adjustment might restore feasbility. If the WBM is feasible on an unconstrained diet two approaches are used to find adjustments to the diet to restore feasibility. 1) All the existing dietary exchange reactions have their lower bounds set to -1000000 to see if increasing metabolites in the existing diet might restore feasibility. If the WBM is feasible by unconstraining the existing diet, the lower bound of each dietary exchange reaction, in an iterative manner, with a lower bound bigger than -0.1 is set to -0.1, 1, and 10. This will identify one by one, if increasing the availability of that metabolite can restore feasibility. 2) The function getMissingDietPersephone.m is used to identify metabolites that could restore feasbility that are not yet part of the existing diet. If any are found, they are added to the diet with a lower bound of -1.

For an indepth tutorial on how to solve WBMs, we refer you to tutorial 2 of Persephone [7]. Here we will further demonstrate how to use WBMs in combination with the nutrition algorithm to predict changes in the diet to achieve desired adjusments in human metabolism.

#### Step 4: Using the nutrition algorithm

We can use either the food item-constrained WBMs or flux-constrained WBMs with the nutrition algorithm to ask for the minimum changes in diet to achieve to biggest change in chosen reactions in human metabolism. Here we will show three scenarios on how to work with the nutrtion algorithm with food-constrained WBM. The same principles apply for flux-constrained WBMs.

The nutrition algorithm has 6 inputs:

- model - A WBM model
- obj - the objective function. Usually this is 'Whole\_body\_objective\_rxn' for WBMs or 'Excretion\_EX\_microbiota\_LI\_biomass[fe]' for community microbiome WBMs.
- objMinMax - if the objective function has to be minimised or maximised. Use either 'min' or 'max', respectively.
- rois - Reactions of interest, a cell array. A list of reactions or metabolites that you want to minimise or maximise and which will be the main drivers to modulate changes in the diet. Metabolites, with their corresponding compartments, will be created as demand reactions. It is important to note that we can at this time only maximimse demand reactions. If existing reactions are picked, double-check the reaction directionality with printRxnFormula.m as it could influence the decision to pick whether to minimise or maximise the reaction.
- roisMinMax - A cell array of the same size as rois. For every reaction of interest chosen, give 'max' or 'min' to maximise or minimise, respectively.
- options - A structure, where every field has an option that modulates how the nutrition algorithm works. We will go over the most relevant options in the various scenarios. For a full list of option please refer to the nutritionAlgorithmWBM.m function description.

And outputs the following:

- newDietModel - A WBM model, with the updated dietary constraints calculated by the algorithm.
- pointsModel - The adjusted model used to arrive to the dietary solution.
- roiFlux - A structure with the solution structures for the original and adjusted diet reaction optimisations of interest .
- pointsModelSln - The solution structure for the optimisation that found the dietary changes.
- menuChanges - A table with the changes in diet food items or dietary metabolites as printed on the terminal.
- macroChanges - A table with the original and new macronutrient distrubutions and the relative changes between the two.
- roiChanges - A table with the range of the fluxes carried by the reactions of interest before and after dietary changes, as printed in the terminal during the run.

We shall use the food-constrained model created in step 3. Let us assume that we want to find dietary alterations that will result in an a) increase of glucose the in blood, b) increase dopamine in the cerebrospinal fluid, and c) minimise the total lipid intake.

If we only set these constraints on the system, we will get unrealistic results as we have not set any bounds on how much food is allowed to be removed or added ,or what the allowed ranges in macronutrient composition are. Also, it may happen that the nutrition algorithm will output unrealistic dietary considerations without setting further details (e.g., to optimise dopamine levels, a recommendation may be to supplement a diet by 9kg of egg whites). Therefore, to ensure obtaining realistic dietary changes, we shall use the (optional) "options" variable to constrain our system. There are many options we can set, but the most relevant for us are:

- options.foodOrMets - indicates if the dietary changes will be calculated through food items or dietary metabolites. Accepted inputs are 'Food', 'allMets', and 'dietMets'. Food will enable food item calculations. allMets will enable calculation of all dietary metabolites. dietMets will enable calculation of metabolites only found in the the given diets. The default is set to 'Food'.
- options.roiWeights - numeric array. Indicates the 'importance' of a reaction of interest. The higher the value, the more the dietary solution will be focused on the minimisation or maxisimation of that particular reactions. By default all reactions of interest are weighted with 10.
- options.caloricRange - a 1x2 numeric array. Specifies what the minimum and maximum range of the consumed calories are allowed to be. It is important to know what the original caloric content of the diet is. If calories of the current diet do not fall within the range you set here, the dietary changes will be altered to fit inside that range. Defaults to [0 1e6].
- options.carbohydrateRange - a 1x2 numeric array. Specifies what the minimum and maximum range of the consumed carbohydrates are allowed to be. It is important to know what the original carbohydrate content of the diet is. If the carbohydrates of the current diet do not fall within the range you set here, the dietary changes will be altered to fit inside that range. Defaults to [0 1e6].
- options.lipidRange - a 1x2 numeric array. Specifies what the minimum and maximum range of the consumed lipids are allowed to be. It is important to know what the original lipid content of the diet is. If the lipids of the current diet do not fall within the range you set here, the dietary changes will be altered to fit inside that range. Defaults to [0 1e6].
- options.proteinRange - a 1x2 numeric array. Specifies what the minimum and maximum range of the consumed proteins are allowed to be. It is important to know what the original protein content of the diet is. If the proteins of the current diet do not fall within the range you set here, the dietary changes will be altered to fit inside that range. Defaults to [0 1e6].
- options.sugarsRange - a 1x2 numeric array. Specifies what the minimum and maximum range of the consumed sugars are allowed to be. It is important to know what the original sugar content of the diet is. If the sugars of the current diet do not fall within the range you set here, the dietary changes will be altered to fit inside that range. Important to note is that the lower bound set for sugars CANNOT be higher than the upper bound of the carboyhydrate range. Defaults to [0 1e6].
- options.foodAddedLimit - A numeric value, specifies how many units of an item (metabolite or food) are allowed to be added to the diet. For food items the unit is in grams, for metabolites it is in mmol. Defaults to 1e6.
- options.foodRemovedLimit - A numeric value, specifies how many units of an item (metabolite or food) are allowed to be removed from the diet. For food items the unit is in grams, for metabolites it is in mmol. Defaults to 1e6.
- options.removeFoodItem: A nx2 cell array with the first column being the food item ID and the second column the name of the database of the food items that shall be removed from the analysis.
- options.addPrice: a nx3 cell array with the first column being the food item ID, the second column the name of the database, and the third column the price (any currency) associated with that food item. Allows for optimising the price of a diet, but only if enough information is available.

These constraints, especially the macronutrient ranges, are only interesting when looking at food item-constrained WBMs as metabolite dietary changes will not take those into account. In theory, it is be possible to set the macronutrient range contraints on flux-constrained WBMs where you calculate additional food items. However, the ranges have to be lower as you can only calculate the added macronutrients from the new food items.

Now that we know the various inputs and constraints we need to take into account let us put it into practice.

% Load in a food item-constrained WBM. Change to where the file is on your

% computer.

model = load('C:\nutritionToolbox\foodDietWBMs\Diet1\_fWBM\_male.mat');

IMPORTANT: To get accurate results, it is imperative that you ensure that the lower and upper bounds of the dietary reactions or food exchange reactions, whichever you use, are the same. If the upper bound is set to 0 or to a higher amount than the lower bound (they are all negative values), the correct macronutrient composition or metabolite composition will not be captured by the model as it can choose to consume less. That will lead to results that only add new foods or metabolites without telling you that it actually consumed less of a particular item. To easily set the upper and lower bounds to the same value use:

for food item-constrained WBMs

model.ub(contains(model.rxns, 'Food\_EX\_')) = model.lb(contains(model.rxns, 'Food\_EX\_'));

for flux-constrained WBMs

% Find all the dietary exchange reactions

dietaryMetExchanges = model.rxns(contains(model.rxns, 'Diet\_EX\_'));

% Remove the dietary exchange of macronutrients as we want those to be

% opened fully

dietaryMetExchanges(strcmp(dietaryMetExchanges, 'Diet\_EX\_energy[d]')) = [];

dietaryMetExchanges(strcmp(dietaryMetExchanges, 'Diet\_EX\_carbohydrate[d]')) = [];

dietaryMetExchanges(strcmp(dietaryMetExchanges, 'Diet\_EX\_protein[d]')) = [];

dietaryMetExchanges(strcmp(dietaryMetExchanges, 'Diet\_EX\_lipid[d]')) = [];

dietaryMetExchanges(strcmp(dietaryMetExchanges, 'Diet\_EX\_sugars[d]')) = [];

dietaryMetExchanges(strcmp(dietaryMetExchanges, 'Diet\_EX\_money[d]')) = [];

% Find the index in the model for the remaining dietary exchange reactions

rxnIdx = findRxnIDs(model, dietaryMetExchanges);

% Set the lower and upper bound to the same value

model.ub(rxnIdx) = model.lb(rxnIdx);

Then we cann set the remaining variables

% Set the objective function to the whole body objective reaction. It does

% not matter if we maxisimise or minimise as the reaction is constrained to

% [1,1].

obj = 'Whole\_body\_objective\_rxn';

objMinMax = 'max';

% Set the reactions of interest and if they have to minimised or maximised

rois = {'glc\_D[bc]', 'dopa[csf]', 'Diet\_EX\_lipid[d]'};

roisMinMax = {'max', 'max', 'min'};

% We want to calculate food item dietary changes

options.foodOrMets = 'Food';

% We will not weight our reaction of interest more then our dietary

% reactions

options.roiWeights=1\*ones(1,length(rois));

First we will show what happens when we do not use any additional dietary constraints on the system.

[newDietModel,pointsModel,roiFlux,pointsModelSln,menuChanges, macroChanges, roiChanges] = nutritionAlgorithmWBM(model,obj,objMinMax,rois,roisMinMax,options);

\_\_\_\_\_\_\_\_\_\_\_\_\_\_\_\_\_\_\_\_\_\_\_\_\_\_\_\_\_\_\_\_\_\_\_\_\_\_\_\_\_\_\_\_\_\_\_\_\_\_\_\_\_
Reaction of Interest 1 = DM\_glc\_D[bc]
Reaction of Interest 2 = DM\_dopa[csf]
Reaction of Interest 3 = Diet\_EX\_lipid[d]
Solution points =-4030.2249
808.4813 come from diet
-4838.7062 come from roi
Food items of interest are:
**foodNames** **status** **Flux** **foodID** **databaseName**
**\_\_\_\_\_\_\_\_\_\_\_\_\_\_\_\_\_\_\_\_\_\_\_\_\_\_\_\_\_\_\_\_\_\_\_\_\_\_\_\_\_\_\_\_\_\_\_\_\_\_\_\_\_\_\_\_\_\_\_\_\_\_\_\_\_\_\_\_\_\_\_\_\_\_\_\_\_** **\_\_\_\_\_\_\_\_\_** **\_\_\_\_\_\_\_\_** **\_\_\_\_\_\_\_\_\_\_\_** **\_\_\_\_\_\_\_\_\_\_\_\_**
{["Alcoholic beverage, distilled, all (gin, rum, vodka, whiskey) 100 proof"]} {'Added'} -0.39132 {'173664' } {'usda' }
{["Cherries, sweet, dark red, raw" ]} {'Added'} -38.092 {'2346399'} {'usda' }
{["Eggs, chicken, egg white, dried" ]} {'Added'} -167.74 {'225' } {'frida'}
{["Sorbitol" ]} {'Added'} -429.33 {'1351' } {'frida'}
{["Linseed oil" ]} {'Added'} -31.608 {'1418' } {'frida'}
{["Lactose, dry, powder" ]} {'Added'} -141.32 {'1680' } {'frida'}
Diet Energy = 4651.9123
f1 =1 & f2=1
DM\_glc\_D[bc]
Original Diet RoI range = 0:1328.4361
New Diet RoI range = 0:4924.7874
DM\_dopa[csf]
Original Diet RoI range = 0:10.7923
New Diet RoI range = 0:94.2549
Diet\_EX\_lipid[d]
Original Diet RoI range = 71.282:71.282
New Diet RoI range = 102.7722:103.4084
\_\_\_\_\_\_\_\_\_\_\_\_\_\_\_\_\_\_\_\_\_\_\_\_\_\_\_\_\_\_\_\_\_\_\_\_\_\_\_\_\_\_\_\_\_\_\_\_\_\_\_\_\_\_\_\_\_\_\_\_\_\_\_\_\_\_\_

As we can see from the printed output, the food items that were added were: Alcoholic beverages, cherries, egg whites, sorbitol, cod liver oil and linseed oil and dried lactose powder. The total amount of food added to the diet is ~810g, which on top of an already normal diet is quite some extra food. When we look further in the dietary composition, 170g of egg white and 141g of dried lactose powder does not seem appetising to eat and difficult to incorporate in an already existing diet. This further asks for additional bounds on food intake and macro-nutrient composition. Interestingly, we see an increase of almost 30g of lipids in the diet, while we asked for the lipid conusmption to be as small as possible. This is likely because we asked for the maximal storage of glucose in the blood. That means that the points generated by adding more glucose in the blood outweigh the points generated to by limiting lipid consumption. This shows to highlight that various reactions of interest can counteract each other as long if one reaction can generate vastly more points than other reactions. A way to counteract this is by adjusting the reaction weights which we show in a section further down.

We do see that the maximum storage of glucose is blood is increased with ~3000 mmol/person/day, the dopamine in the cerebral spine fluid with ~85 mmol/person/day and consumed lipids with 33 g/person/day. The changes in diet, macronutrient composition, and reaction of interest fluxes can be found in the output variables dietChanges, macroChanges, and roiChanges, respectively.

To obtain a more palatable dietary change we will now add additional constraints to food consumed and macronutrient composition.

From the diet analysis performed in step 3, we see that we have ~345g of carbohydrates, ~45g of proteins, ~70g of lipids, ~195g of sugar and ~2200 kcal (found in the macrosAsReported.csv table). Depending on your modelling objectives, you can use these values as a starting point to set constraints. If you want the diet to remain in a similar range of macronutrient composition, we suggest to have the constraints 10-20% of the original value to allow for some leeway. Otherwise, if you want to drastically alter the composition, you can adjust the constraints however you like it. You may also choose to adjust only one or two macronutrient constraints. Just be aware that diet will only be as realistic as the constraints you put on the system.

Here we will set the constraints to keep more or less the same dietary composition, and we shall only add 150g of new food items at maximum. We also demonstrate here the capability to remove food items from the analysis. We saw in the previous calculation that we should add 130g of sorbitol to the diet, an item that is not typically consumed in its raw form. Here we remove the item with the option "removeFoodItems". To discard further items from analysis, add new rows with ;.

% Let us clear the options from the previous run

clear options

% Set the new options for the new run

% We want to calculate food item dietary changes

options.foodOrMets = 'Food';

% We will not weight our reaction of interest more than our dietary

% reactions

options.roiWeights=[1 1 1];

% Set the dietary limits

options.caloricRange=[1900 2300];

options.carbohydrateRange = [300 370];

options.lipidRange = [60 80];

options.proteinRange = [40 60];

options.sugarsRange = [170 210];

% Set the maximum amount of food addded

options.foodAddedLimit=150;

% Remove food item sorbitol

options.removeFoodItem = {'1351', 'frida'};

% Run the nutrition algorithm again

[newDietModel,pointsModel,roiFlux,pointsModelSln,menuChanges, macroChanges, roiChanges] = nutritionAlgorithmWBM(model,obj,objMinMax,rois,roisMinMax,options);

\_\_\_\_\_\_\_\_\_\_\_\_\_\_\_\_\_\_\_\_\_\_\_\_\_\_\_\_\_\_\_\_\_\_\_\_\_\_\_\_\_\_\_\_\_\_\_\_\_\_\_\_\_
Reaction of Interest 1 = DM\_glc\_D[bc]
Reaction of Interest 2 = DM\_dopa[csf]
Reaction of Interest 3 = Diet\_EX\_lipid[d]
Solution points =-2817.1291
180 come from diet
-2997.1291 come from roi
Food items of interest are:
**foodNames** **status** **Flux** **foodID** **databaseName**
**\_\_\_\_\_\_\_\_\_\_\_\_\_\_\_\_\_\_\_\_\_\_\_\_\_\_\_\_\_\_\_\_\_\_\_\_\_\_\_\_\_\_\_\_\_\_\_\_\_\_\_\_\_\_\_\_\_\_\_\_\_\_\_\_\_\_\_\_\_\_\_\_\_\_\_\_\_** **\_\_\_\_\_\_\_\_\_\_\_** **\_\_\_\_\_\_\_** **\_\_\_\_\_\_\_\_\_\_\_** **\_\_\_\_\_\_\_\_\_\_\_\_**
{["Alcoholic beverage, distilled, all (gin, rum, vodka, whiskey) 100 proof"]} {'Added' } -2.0068 {'173664' } {'usda' }
{["Oil, soybean" ]} {'Added' } -11.973 {'748366' } {'usda' }
{["Oil, olive, extra light" ]} {'Added' } -15.159 {'1750351'} {'usda' }
{["Cherries, sweet, dark red, raw" ]} {'Added' } -72.096 {'2346399'} {'usda' }
{["Eggs, chicken, egg white, dried" ]} {'Added' } -16.928 {'225' } {'frida'}
{["Palm kernel oil" ]} {'Added' } -27.356 {'1377' } {'frida'}
{["Lactose, dry, powder" ]} {'Added' } -4.4806 {'1680' } {'frida'}
{["Oil, rape seed (no eruca acid)" ]} {'Removed'} -30 {'1483' } {'frida'}
Diet Energy = 2300
f1 =1 & f2=1
DM\_glc\_D[bc]
Original Diet RoI range = 0:1328.4361
New Diet RoI range = 0:3081.9782
DM\_dopa[csf]
Original Diet RoI range = 0:10.7923
New Diet RoI range = 0:19.2152
Diet\_EX\_lipid[d]
Original Diet RoI range = 71.282:71.282
New Diet RoI range = 79.6128:80.3872
\_\_\_\_\_\_\_\_\_\_\_\_\_\_\_\_\_\_\_\_\_\_\_\_\_\_\_\_\_\_\_\_\_\_\_\_\_\_\_\_\_\_\_\_\_\_\_\_\_\_\_\_\_\_\_\_\_\_\_\_\_\_\_\_\_\_\_

We can see that the dietary changes are different, although some items remained the same. We now add ~150g of food to the diet while removing ~30g of food from the diet. The removed food was likely removed to make space for the carbohydrate macronutrient constraints. We can also see that, indeed, sorbitol does no longer show up in the solution, as we removed it in the options. To double-check if it would show up in the solution with these new constraints you can comment out (put % in front of) the line where we set which food items we want to have removed and rerun.

The main difference we see in our reactions of interest is for dopamine in the cerebrospinal fluid, where we now only see an increase of ~9 mmol/person/day. A possbile explanation is that the solution in the nutrition algorithm is biased by how many points are generated by the reactions of interest. It sees that more points can be achieved with glucose (up to ~3000 points), which is far higher than for dopamine. This leads to the algorithm preferring dietary changes to favour increased glucose in the blood, as this will garner more points. A way to combat this bias is by adjusting the weight of the dopamine reaction in roiWeights option to 10 or 100, so that every point made in that reaction will be counted 10 or 100 times as much. This way the reactions of interest can have the same influence on the solution.

If we look at the macronutrient distribution:

disp(macroChanges);

**macroNames** **originalMacro** **newMacro** **relChange**
**\_\_\_\_\_\_\_\_\_\_\_\_\_\_\_\_** **\_\_\_\_\_\_\_\_\_\_\_\_\_** **\_\_\_\_\_\_\_\_** **\_\_\_\_\_\_\_\_\_**
{'energy' } 2190.8 2301.2 110.36
{'carbohydrate'} 344.36 361.37 17.019
{'protein' } 45.317 59.853 14.536
{'lipid' } 71.282 80.384 9.1024
{'sugars' } 194.9 209.85 14.954

We see indeed that all the new macronutrients are within the ranges we set. It might slighty pass over or under the set bounds as we set the diet with a 1% margin of the calculated optimal dietary changes. We also see that despite asking to minimise the lipids in the diet, it still calculated that the optimal solution would be to add the highstest allowed amount of lipids. This means that its more important for the model to get the glucose maximised than it is to minimise the lipid consumption. Again we change the roiWeights to give more importance to minimising the lipid content in the diet to limit the effect glucose has on the solution.

To give the diets an addtional layer of realism, we can add on the money food items costs. This can be done through the options variable:

options.addPrice: a nx3 cell array with the first column the food item ID, the second column the name of the database, and in the third column the price (any currency) associated with that food item. This allows for optimising the cost of a diet, but only if sufficient information is available.

Important is that you remove all other food items that you do not have price information for. Otherwise the algorithm will include those items as they "do not cost anything". Note that this will be a time-intensive option as you will have to link price to the various database items of interest.

This concludes the tutorial on how to work with the nutrition toolbox. If more information is required on the nutrition algorithm, we recommened reading the original paper [2] and looking at the tutorial on just the nutrition algorithm, present in the COBRA toolbox. For any questions, please use the COBRA toolbox google community https://groups.google.com/g/cobra-toolbox , the issues on the COBRA toolbox github, or contact us directly through the corresponding author from the publication associated with this tutorial.

## REFERENCES

1. U.S. Department of Agriculture, Agricultural Research Service, Beltsville Human Nutrition Research Center. FoodData Central. Available from https://fdc.nal.usda.gov/.

2. Food data (frida.fooddata.dk), version 5.3, 2024, National Food Institute, Technical University of Denmark

3. Thiele, I., Sahoo, S., Heinken, A., Hertel, J., Heirendt, L., Aurich, M. K., & Fleming, R. M. (2020). Personalized whole‐body models integrate metabolism, physiology, and the gut microbiome. Molecular systems biology, 16(5), e8982.

4. Weston, B. R., & Thiele, I. (2023). A nutrition algorithm to optimize feed and medium composition using genome-scale metabolic models. Metabolic Engineering, 76, 167-178.

5. Heirendt, L, et al., Creation and analysis of biochemical constraint-based models using the cobra toolbox v.3.0. Nature Protocols, 14(3):639–702, February 2019.

6. National Research Council (US) Committee on Diet and Health. Diet and Health: Implications for Reducing Chronic Disease Risk. Washington (DC): National Academies Press (US); 1989. 6, Calories: Total Macronutrient Intake, Energy Expenditure, and Net Energy Stores. Available from: https://www.ncbi.nlm.nih.gov/books/NBK218769/

7. Nap et al., Persephone: A personalisation and evaluation pipeline for human whole-body metabolic modelsPersephone: A personalisation and evaluation pipeline for human whole-body metabolic models. Submitted.
